# Investigation of proarrhythmic effect of high sugammadex doses: an experimental animal study

**DOI:** 10.1186/s44158-022-00077-0

**Published:** 2022-12-23

**Authors:** Emin Tunç Demir, Mesut Erbaş

**Affiliations:** 1 Department of Anesthesiology and Reanimation, Intensive Care Science, Aydin State Hospital, Aydin, Turkey; 2 Department of Anesthesiology and Reanimation, Çanakkale Onsekiz Mart University Faculty of Medicine, Çanakkale, Turkey

**Keywords:** Sugammadex, QT intervals, Arrhythmia, Electrocardiography, Experimental study

## Abstract

**Background:**

Studies on higher doses of sugammadex effect on QT interval and leading arrhythmia have been limited. In this study, we aimed to investigate possible proarrhythmic effect of higher doses of sugammadex in conditions that required urgent reversal of neuromuscular blockade during general anesthesia in an experimental animal model.

**Methods:**

It was experimental animal study. Total of 15 male New Zealand rabbits were randomly divided into three groups for low (4 mg/kg, *n* = 5), moderate (16 mg/kg, *n* = 5), and high dose of sugammadex (32 mg/kg, *n* = 5). All rabbits were premedicated by intramuscular ketamine 10 mg/kg, and general anesthesia was inducted by intravenous injection of 2 mg/kg of a propofol, 1 mcg/kg fentanyl, and 0.6 mg/kg rocuronium injection. Airway was provided by V-gel rabbit and connected to anesthetic device and ventilated at about 40 cycle/min and 10 ml/kg; oxygen 50% plus air 50% mixture was used with 1 MAC isoflurane to maintain anesthesia. Electrocardiographic monitorization and arterial cannulation were provided to follow-up mean arterial pressure and for arterial blood gas analyses. Intravenous sugammadex in three different doses were injected at 25th min of induction. After observing adequate respiration of all rabbits, V-gel rabbit was removed. Parameters and ECG recordings were taken basal value before induction and at the 5th, 10th, 20th, 25th, 30th, and 40th min to measure corrected QT intervals and were stored on digital media. QT interval was calculated as the time from the beginning of the Q wave to the end of the T wave. Corrected QT interval was calculated according to the Bazett’s formula. Possible adverse effects were observed and recorded.

**Results:**

In all three groups, there was no significant statistical difference in mean arterial blood gases parameters, arterial pressures, heart rates, and Bazett QTc values, and no serious arrhythmia was recorded.

**Conclusion:**

We found in animal study that low, moderate, and high doses of sugammadex did not significantly altered corrected QT intervals and did not cause any significant arrhythmia.

## Background

General anesthesia is characterized by five main effects as those temporary loss of consciousness, amnesia, analgesia, inhibition of autonomic reflexes, and skeletal muscle relaxation [1]. Neuromuscular blocking agents (NMBA) are commonly used during general anesthesia to facilitate tracheal intubation, to maintain mechanical ventilation, and to optimize patients’ condition for surgical procedure [2]. Rocuronium bromide and vecuronium bromide are the most frequently used NMBAs [2]. Afterwards, a successful reversal of neuromuscular blockage, and avoiding postoperative rebounds, are also important. Neostigmine is the most frequently used pharmacological agent to eliminate the effects of NMBAs, however, itself causes some side effects [3, 4]. Especially, NMBA antagonists neostigmine and edrophonium, and anticholinergics such as atropine and glycopyridine, have been reported to prolong QT intervals [5].

QT interval refers to the duration of the ventricular depolarization and repolarization when the ventricular muscle is at refractory stage. In electrocardiography (ECG) recordings, it is calculated by the length between beginning of QRs complex to the end of the T wave. Therefore, QT interval is directly affected by heart rate, and a correction by heart rate is required for standardization that is called as corrected QT interval (QTc). It is known that prolongation of QT interval increases the risk of serious ventricular arrhythmias, including ventricular fibrillation and polymorphic ventricular tachycardia [6, 7]. QTc interval prolongation has been shown to be associated with sudden cardiac death especially in patients with ischemic heart disease and even in healthy individuals [8, 9]. There are some factors that prolong QT interval, and drugs are encountered as the most important causes [10]. Many of agents that are used in the practice of anesthesia affect QT interval. In recent years, there have been researches investigating the effects of anesthetics agents on QT interval [11–13]. Prolonged QT intervals have been associated with life-threatening arrhythmias and sudden deaths in patients under general anesthesia [14, 15].

Sugammadex, biologically inactivated analog of cyclodextrin, is a fast, safe, and well-tolerated agent that eliminates effects of nondepolarizing NMBAs [16]. Preliminary sugammadex studies on healthy individuals have reported no significant adverse effects on blood pressure, heart rate, and respiratory parameters [17, 18]. The recommended dosage for clinical use is 2–16 mg/kg, reported to be safe [19]. McDonnell et al. reported a rapid and successful reversal of rocuronium related anaphylaxis with a high dose of sugammadex [20].

In patients with unstable hemodynamic conditions such as trauma/emergency surgery that perioperative period is expected to be challenging, a faster elimination of NMBAs is desired. In those situations, higher doses of sugammadex (up to 32 mg/kg) use could be useful considering its safe and feasible hemodynamic profile. In this study, we aimed to evaluate safety profile of sugammadex in higher doses (32 mg/kg) in terms of hemodynamic status, QTc variations, and arrhythmias. An experimental animal general anesthesia model, without any surgical stimulus, was set to compare effects of low (4 mg/kg) (L), medium (16 mg/kg) (M), and higher doses (32 mg/kg) (H) of sugammadex.

## Methods

This study was conducted in a university-based animal research laboratory, in June 2017. Ethical approval was obtained from local university Animal Research Ethics Committee. The rules on the care and use of laboratory animals in the 1964 Helsinki Declaration were carefully applied throughout the study.

The study was conducted on 15 (male) healthy adult white New Zealand rabbits weighing between 2.5 and 3.5 kg. Animals were fed ad libitum in cages, inhabited in ecological rooms with set temperatures of 21 ± 2 °C, and arranged light/dark cycles of 12/12 h. The rabbits were overnight fasted for 8 h before the procedures, and all procedures were performed between 9:00 and 16:00.

The animals were randomly divided into three groups as those low-dose (group L, *n* = 5), medium-dose (group M, *n* = 5), and high-dose sugammadex groups (group H, *n* = 5).

At the beginning of procedure, intramuscular ketamine hydrochloride 10 mg/kg was administered to animals for premedication, and 20 min of rest was given. Animals were monitored with ECG (Digital ECG system Poly-Spectrum-8/E, Neurosoft Ltd. 5, Voronin str., Ivanovo, Russia), and canulation was provided via marginal auricular vein using a 26-gauge catheter (Bicakcilar Tibbi Cihazlar AŞ, Istanbul, Turkey). Fluid resuscitation was provided as required, and 4 L/min O_2_ was administered through a mask. A 24-gauge catheter (Bicakcilar Tibbi Cihazlar AŞ, Istanbul, Turkey) was used to canulate the central auricular artery to monitorize mean arterial pressure (PETAŞ® KMA 800 monitor device, Ankara, Turkey). After induction of general anesthesia by administering intravenous injection of 2 mg/kg of a propofol, 1 mcg/kg fentanyl, and 0.6 mg/kg rocuronium, V-Gel Rabbit (V-gel rabbit R-3 Docsinnovent(r) Ltd. London, UK) was inserted to provide reliable airway and connected to anesthetic device (Anesthesia Machine w/O2 Flush Model M3000PK Parkland Scientific Lab and Research Equipment, Florida, USA). Animals were manually ventilated by the same anesthetist, with respiration rate of about 40 cycle per minute and with pressure of 15 cmH_2_O (about 10 mL/kg) in convenience with rabbit physiology. Oxygen 50% plus air 50% mixture was used with 1 MAC isoflurane to maintain anesthesia.

Sugammadex at doses of 4 mg/kg intravenous injection for group L, 16 mg/kg intravenous injection for group M, and 32 mg/kg intravenous injection for group H was administered at the 25th min of induction. After the procedure and anesthesia was seized, animals were observed for sufficient spontaneous respiration, V-gel rabbit was removed, and animals were put in rest.

Recordings were taken, and data were collected at just basal value before induction and at the 5th, 10th, 20th, 25th, 30th, and 40th min. Heart rate and mean arterial pressure values were continuously recorded. Arterial blood gases were obtained and analyzed before and at 10 and 40 min after induction (Blood Gas Analyzer — Gastat 600 Series, Techno Medica Co. Ltd., Yokohama, Japan) to evaluate oxygenation status. ECG readings were taken with 1 mV = 20 mm, at 50 mm/s speed, and 35 Hz filter by electrodes placed at extremities to record DI, DII, DIII, aVR, aVL, and aVF derivations, at basal value before induction, and at the 5th, 10th, 20th, 25th, 30th and 40th min after induction, according to the method reported by Uzun et al. [21]. ECG data were stored in and analyzed by a digital device and software (Poly-Spectrum 12 channel ECG-System, Poly-Spectrum-8, Neurosoft, 5, Voronin str., Ivanovo, Russia), which QTc was calculated according to Bazett formula [22]. In assessment, QTc values between 350 and 450 msecs were accepted within normal ranges. A prolonged QTc reading above 450 msecs was deemed as moderate and more than 500 msecs as high risk for arrhythmia.

Statistical analysis of data was performed using Statistical Package for Social Sciences version 20 (SPSS© Inc., Chicago, IL, USA) software. Descriptive data were presented as frequencies, median, minimum, and maximum values, and mean and standard deviations as appropriate. *T*-tests and chi-square tests were processed when required. Statistical significance was taken as *p*-values < 0.05 with confidence intervals of 95%.

## Results

All experimental rabbits, used in three different groups, were healthy, and there was no significant difference in terms of rabbits’ weight between groups.

In all groups, during the 25th min of the study, an increase in mean arterial pressure was observed with the application of sugammadex. It has been evaluated as a decrease in the effect of anesthetic drugs. In statistical comparisons, no significant difference in mean arterial pressures was found between groups.

There was no significant statistical difference, except heart rate significantly increased at the 5th min correlating with higher doses. The increase in heart rate at the 5th min has no significant effect on QT and other clinical parameters (Table 1).

**Table 1:** Presentation of mean heart rate and mean arterial pressure recordings (mean ± SD) for three different sugammadex dose groups through experiment by intervals. There was no significant statistical difference, except heart rate significantly increased at the 5th min correlating with higher doses

	Group L	Group M	Group H	***p***
**Heart rate**				
*Basal*	156.2 ± 53.7	228.4 ± 18.6	196.0 ± 41.2	0.083
*5 min*	153.6 ± 26.1	191.8 ± 18.8	215.2 ± 21.6	**0.008**
*10 min*	161.2 ± 26.0	215.0 ± 23.2	198.8 ± 47.2	0.100
*20 min*	167.8 ± 33.2	201.4 ± 22.6	211.2 ± 33.8	0.174
*25 min*	172.6 ± 18.3	204.2 ± 16.5	200.6 ± 29.2	0.049
*30 min*	164.2 ± 28.5	184.4 ± 27.9	200.0 ± 24.4	0.222
*40 min*	164.2 ± 27.5	179.4 ± 36.1	207.4 ± 31.9	0.156
**Mean arterial pressure**				
*Basal*	71.8 ± 6.4	89.6 ± 21.8	78.4 ± 5.6	0.364
*5 min*	66.2 ± 13.1	73.8 ± 20.8	79.0 ± 18.4	0.562
*10 min*	70.2 ± 1.8	63.8 ± 6.7	74.6 ± 10.4	0.228
*20 min*	61.6 ± 8.4	66.4 ± 9.0	69.8 ± 11.3	0.578
*25 min*	64.2 ± 6.6	85.8 ± 16.4	76.8 ± 10.8	0.061
*30 min*	70.0 ± 11.5	86.0 ± 11.9	75.6 ± 10.7	0.064
*40 min*	78.4 ± 22.0	94.6 ± 13.1	83.2 ± 17.5	0.402

*SD* standard deviation, *p* Kruskal-Wallis test. *L*, low dose (4 mg/kg); *M*, medium dose (16 mg/kg); *H*, high dose (32 mg/kg)

**Fig. 1:**
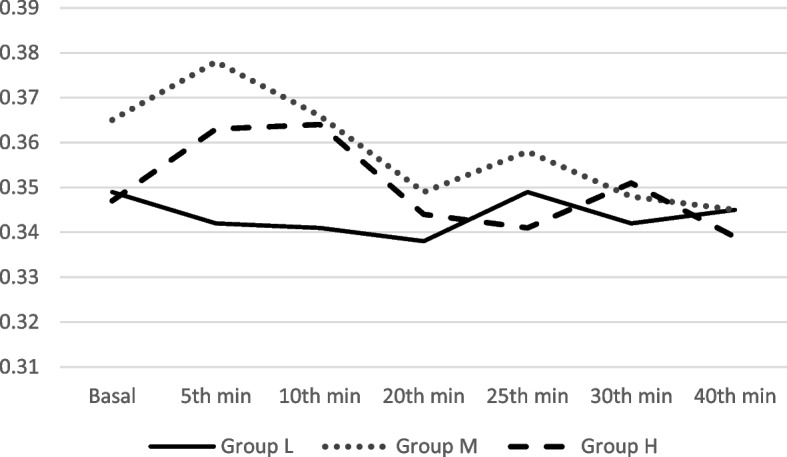
Presentation of mean corrected QT values (mean ± SD) for three different sugammadex dose groups through experiment by intervals, calculated by Bazett method on ECG recordings. L, low dose (4 mg/kg); M, medium dose (16 mg/kg); H, high dose (32 mg/kg)

When group’s basal Bazett QTc values were processed, the average value of group L was 0.349, group M was 0.365, and group H was 0.347. Within groups, analyses showed no significant statistical difference in mean Bazett QTc values, both before and after sugammadex application. The mean Bazett QTc values of different groups were compared, which were measured at the same time; no significant statistical difference was found (Table 2 and Fig. 1).

**Table 2:** Presentation of mean corrected QT values (mean ± SD) for three different sugammadex dose groups through experiment by intervals, calculated by Bazett method on ECG recordings. There was no significant statistical difference by different doses through experiment

	Group L	Group M	Group H	***p***
*Basal*	0.349 ± 0.015	0.365 ± 0.012	0.347 ± 0.016	0.092
*5 min*	0.342 ± 0.007	0.378 ± 0.008	0.363 ± 0.031	0.085
*10 min*	0.341 ± 0.007	0.366 ± 0.017	0.364 ± 0.029	0.093
*20 min*	0.338 ± 0.005	0.349 ± 0.008	0.344 ± 0.016	0.151
*25 min*	0.345 ± 0.016	0.358 ± 0.019	0.341 ± 0.011	0.210
*30 min*	0.342 ± 0.011	0.348 ± 0.011	0.351 ± 0.015	0.552
*40 min*	0.343 ± 0.013	0.345 ± 0.010	0.339 ± 0.009	0.664

*SD* standard deviation, *p* Kruskal-Wallis test. *L*, low dose (4 mg/kg); *M*, medium dose (16 mg/kg); *H*, high dose (32 mg/kg)

Mean pH values were 7.365 for group L, 7.357 for group M, and 7.355 for group H, which were not significantly different. In addition, mean values of the other arterial blood gas parameters were also not statistically different for all groups (Table 3).

**Table 3:** Presentation of mean arterial blood gases parameter recordings (mean ± SD) for three different sugammadex dose groups through experiment. There was no significant statistical difference

	Group L	Group M	Group H	***p***
**pH**	7.365 ± 0.01	7.357 ± 0.02	7.355 ± 0.02	> *0.05*
**PO**_**2**_ **(mmHg)**	136.4 ± 18.8	126.9 ± 22.4	122.5 ± 24.6	
**PCO**_**2**_ **(mmHg)**	35.7 ± 3.1	36.6 ± 1.3	36.3 ± 4.3	
**HCO**_**3**_ **(mmol/L)**	24.0 ± 1.3	23.3 ± 0.6	23.0 ± 0.7	

*SD* standard deviation, *p* Kruskal-Wallis test. *L*, low dose (4 mg/kg); *M*, medium dose (16 mg/kg); *H*, high dose (32 mg/kg)

Detailed data were presented in supplementary materials, tables, and figures.

## Discussion

We did not perform a sham surgical procedure in our experiment, in order to gain a more precise evaluation of sugammadex effects, since sympathetic activation by a surgical stimulus could cause tachycardia, vasoconstriction, unpredictable blood pressure variations, and possibly arrhythmias. Using the model, we compared the different effects of low (4 mg/kg), medium (16 mg/kg), and a higher (32 mg/kg) dose of sugammadex and its effects on QT duration.

It has been shown that many drugs used in anesthesia practice can affect the QT interval. The idea that QT interval may have an impactful correlation to the life-threatening arrhythmias and sudden death cases during anesthesia application has been highlighted [23]. As a result, many studies have been conducted to investigate the effect of many anesthetic drugs on the QT interval [24–26].

When general anesthesia is applied to patients with an undiagnosed congenital or acquired long QT interval, ventricular arrhythmia, ventricular tachycardia, ventricular fibrillation or torsade de pointes (TdP) phenomenon, even sudden death may occur [14, 27]. The importance of continuous ECG monitoring during anesthesia and thoroughly evaluating the patient’s cardiac rhythm analysis before anesthesia is increasing due to the inclusion of many drugs in anesthesia studies that research prolongation in QT and QTc values [28].

Muscle relaxant application, which is an important parameter of general anesthesia, is routinely used for balanced anesthesia [2]. Among the steroid NMBAs, rocuronium and vecuronium are the most commonly used muscle relaxant agents. Cholinesterase inhibitors are traditionally used to reverse neuromuscular blockade. Neostigmine is the most potent and selective of these agents. However, when neostigmine is used alone, it causes side effects such as bradycardia, QT prolongation, bronchoconstriction, hypersalivation, and increased motility, which concern many systems, so anticholinergic agents such as atropine and glycopyrrolate are used to prevent these side effects [3, 4]. Neostigmine, edrophonium, and the anticholinergic agents atropine and glycopyrrolate have also been reported to prolong the QT interval [29]. Today, sugammadex is an alternative to the traditionally applied cholinesterase inhibitors in recurrence protocols. With the administration of sugammadex, selectively developed to rocuronium and vecuronium, postoperative residual curarization and muscarinic side effects are not expected [30, 31]. It has also been shown to provide good cardiovascular stability [32]. In studies in healthy patients, sugammadex, effects on blood pressure, heart rate, respiration, and thermoregulation were not clinically significant [33]. In their study, Kara et al. [34] evaluated the effectiveness of sugammadex and neostigmine in terms of cardiovascular stability. Sugammadex did not appear to have a significant effect on heart rate. A significant increase in heart rate was detected 5 and 10 min after the application of neostigmine. In the study conducted by Woo et al. [35], no clinically significant difference was found between the treatment groups in systolic, diastolic, and mean arterial pressure (MAP). In our study, no statistically significant difference was found between the groups in which different doses of sugammadex were administered, in terms of mean heart rate measurements, confirming the literature. No statistically significant difference was found between the groups in terms of MAP averages, confirming that cardiovascular stability was not impaired. In trauma and emergency surgical procedures where cardiovascular stability is important, it is important that sugammadex does not cause effects such as hypotension and bradycardia even when used in high doses.

In the study of De Kam et al. [26], investigating the effects of different doses of sugammadex on QTc prolongation, ventricular tachycardia was detected in only one case 4 h after sugammadex administration, but its relationship with the drug was not clear. In Dahl et al.’s [36] study searching the safety and efficacy of sugammadex in reversing neuromuscular blockage in the cardiac patient population who undergone noncardiac surgery, 116 patients were selected, who were classes 2–3 according to the New York Heart Association classification. The patients were divided into 3 groups: the placebo group, 2 mg/kg sugammadex group, and 4 mg/kg sugammadex group. The study compared the mean systolic and diastolic blood pressure between placebo and sugammadex groups; it was found to be higher in the sugammadex groups. Heart rate remained stable after administration of sugammadex and placebo. QTc was evaluated by two different methods, and there was no significant difference in QTc values, while moderate QTcB prolongation was observed in only 1 case. In our study, sugammadex was administered during the 25th min on every group in specified doses. There was no significant difference in QTc measurements even in higher doses.

The recommended drug doses of sugammadex for use in clinical practice are indicated as 2–16 mg/kg. However, there are case reports and publications about the use of high doses in the literature. In their case report, McDonnell et al. [20] showed that sugammadex was administered in a high dose (500 mg) for anaphylaxis to rocuronium, consequently reversed anaphylactic shock state, and ameliorated all vital signs of the patient. In a case report published by Molina et al. [37] involving the reversal of deep neuromuscular block caused by rocuronium with a mistakenly applied very high dose (40 mg/kg) sugammadex, as we examined in our Group H, there was no clinical change in blood pressure, heart rate, or ECG. Peeters et al. [38] aimed to evaluate pharmacokinetics, safety, and tolerance by using high doses of sugammadex (up to 96 mg/kg) in their study on healthy individuals. When sugammadex was given at a dose higher than the recommended dose in only one patient, side effects related to hypersensitivity, such as an unpleasant taste in the mouth and sporadic cough, were observed, and changes in vital signs and QTc measurements were not detected in other volunteers.

Kam et al. [26] researched the effects of sugammadex doses of up to 32 mg/kg and combination of sugammadex and steroid neuromuscular agents on QTc prolongation on healthy individuals, as in our experiment, and they found no difference. The common result of our and these studies is that there is no significant difference between the measured QTc values with different doses of sugammadex, even with higher doses than recommended. On the contrary, the effects of medications used during surgeries requiring general anesthesia cannot be fully evaluated, because traumatic state caused by surgery itself has some consequences on cardiovascular system. Our study is the first experimental study evaluating the efficacy and safety of different consequent doses of sugammadex on QT interval time.

Studies have shown that many of the drugs used in anesthesia practice may cause QTc prolongation; therefore, an utmost importance should be given to preanesthetic evaluation of trauma, and emergency surgery patients predisposed to QT, QTc, and QTd prolongations. In addition, ECG monitoring during anesthesia holds a great importance.

In anesthesia practice, abnormally prolonged QT will make heart susceptible to dysrhythmias, especially in patients with history of trauma, emergency surgery patients, and cardiovascular diseases. As the results of our study show, different doses of sugammadex given to subjects under general anesthesia did not cause a difference on QTc duration, even in higher doses than recommended. The results of our study are similar to other few studies regarding the topic, and we think that further studies are required for sugammadex to be used at high doses in patients with a history of trauma, emergency surgery patients, and cardiovascular diseases.

## Conclusion

We found in animal study that low, moderate, and high doses of sugammadex did not significantly altered corrected QT intervals and did not cause any significant arrhythmia and showed that sugammadex in higher doses could be safely used when urgent reversal of NMBAs is required. Further feasibility studies are required to evaluate use of higher doses of sugammadex.

## Data Availability

The datasets used and/or analyzed during the current study are available from the corresponding author on reasonable request.

## Abbreviations

NMBA: Neuromuscular blocking agents
ECG: Electrocardiography
QTc: Corrected QT interval
